# Determination of influenza B identity and potency in quadrivalent inactivated influenza vaccines using lineage-specific monoclonal antibodies

**DOI:** 10.1371/journal.pone.0175733

**Published:** 2017-04-19

**Authors:** Swati Verma, Jackeline Soto, Anupama Vasudevan, Falko Schmeisser, Esmeralda Alvarado-Facundo, Wei Wang, Carol D. Weiss, Jerry P. Weir

**Affiliations:** 1 Laboratory of DNA Viruses, Division of Viral Products, Center for Biologics Evaluation and Research, Food and Drug Administration, Silver Spring, MD, United States of America; 2 Laboratory of Immunoregulation, Division of Viral Products, Center for Biologics Evaluation and Research, Food and Drug Administration, Silver Spring, MD, United States of America; Icahn School of Medicine at Mount Sinai, UNITED STATES

## Abstract

Co-circulation of two antigenically and genetically distinct lineages of influenza B virus, represented by prototype viruses B/Victoria/2/1987 and B/Yamagata/16/1988, has led to the development of quadrivalent influenza vaccines that contain two influenza B antigens. The inclusion of two influenza B antigens presents challenges for the production and regulation of inactivated quadrivalent vaccines, including the potential for cross-reactivity of the reagents used in identity and potency assays because of the relative close relatedness of the hemagglutinin (HA) from the two virus lineages. Monoclonal antibodies (mAbs) specific for the two lineages of influenza B HA were generated and characterized and used to set-up simple identity tests that distinguish the influenza B antigens in inactivated trivalent and quadrivalent vaccines. The lineage-specific mAbs bound well to the HA of influenza B strains included in influenza vaccines over a period of more than 10 years, suggesting that identity tests using such lineage-specific mAbs would not necessarily have to be updated with every influenza B vaccine strain change. These lineage-specific mAbs were also used in an antibody capture ELISA format to quantify HA in vaccine samples, including monovalent, trivalent, and quadrivalent vaccine samples from various manufacturers. The results demonstrated correlation with HA values determined by the traditional single radial immunodiffusion (SRID) assay. Further, the antibody-capture ELISA was able to distinguish heat-stressed vaccine from unstressed vaccine, and was similar to the SRID in quantifying the resultant loss of potency. These mAb reagents should be useful for further development of antibody-based alternative influenza B identity and potency assays.

## Introduction

Influenza continues to be a major infectious disease threat worldwide, causing seasonal epidemics, and occasional pandemics due to the emergence of an influenza A virus containing a hemagglutinin (HA) subtype that has not recently circulated in humans. Two influenza A subtypes, H1N1 and H3N2, have co-circulated in humans since 1977. In addition, influenza B is also responsible for seasonal influenza epidemics. Since influenza B viruses belong to a single subtype and do not have animal reservoirs like influenza A, they are unable to initiate a pandemic. Nevertheless, influenza B infection causes significant human disease resulting in absenteeism, hospitalizations and even death. Influenza B virus infections average between 20–30% of the total influenza cases each year [1]. Although all influenza B viruses are classified as a single influenza subtype, two antigenically and genetically distinct lineages, represented by prototype viruses B/Victoria/2/1987 (Victoria lineage) and B/Yamagata/16/1988 (Yamagata Lineage), have co-circulated since 1983 [2]. The low level of cross-protection provided by immunization with vaccine containing antigen from a single influenza B lineage indicated the need for development of quadrivalent vaccines containing influenza A H1N1 and H3N2 antigens and influenza B antigens from both lineages [3, 4]. Several quadrivalent influenza vaccines are now licensed in the United States [5].

The inclusion of antigens from both influenza B lineages presents challenges for the production and regulation of quadrivalent vaccines, especially in light of the restrictive timelines of seasonal vaccine production. Not only are influenza B vaccine viruses relatively poor growing compared to high growth influenza A vaccine viruses, but two sets of influenza B potency reagents are needed for formulation, both to quantify each HA antigen present and to verify the identity of each antigen in the vaccine. The cross-reactivity of influenza B reagents in identity and potency assays is always a potential issue because of the relative close relatedness of the HAs from the two influenza B lineages.

Since the 1970’s, the potency and identity of inactivated influenza vaccines has been determined by using the single radial immunodiffusion (SRID) assay [6–8]. The assay is simple, accurate and does not require sophisticated instruments. Moreover, the potency value determined by SRID correlates with immunogenicity [9–12] which correlates with clinical benefit [13]. Nevertheless, limitations of the assay, such as the dynamic range and the need for large amounts of standardized reagents [14], have indicated the need for the development of alternative methods to quantify HA in inactivated influenza vaccines. Several assays have now been described that show feasibility for further development [15–18], including multiple approaches and platforms that use monoclonal antibodies (mAbs) to capture and quantify HA in vaccine samples [19–22].

Here we describe the generation and characterization of mAbs that are specific for the two lineages of influenza B HA. We demonstrate that these lineage-specific mAbs can be used to set-up simple identity tests that distinguish the influenza B antigens in inactivated influenza vaccines, including quadrivalent vaccines, which can be problematic with the SRID assay due to the potential for cross-reactivity of the polyclonal antiserum used in the assay. We also use these mAbs in an antibody capture ELISA format to quantify HA in vaccine samples, demonstrating correlation with HA values determined by traditional SRID, and the ability to distinguish and quantify sub-potent vaccine stressed by heat-treatment. These reagents should be useful for further development of antibody-based alternative influenza potency assays.

## Materials and methods

### Cells and viruses

Influenza vaccine viruses were propagated in 9-day-old specific pathogen-free embryonated chicken eggs. Selection of escape mutant influenza viruses was performed in MDCK cells [23]. Modified vaccinia virus Ankara (MVA) vectors expressing influenza hemagglutinin (HA) have been described previously [24, 25] and were propagated and titered in DF-1 cells. All methods for the generation, propagation, and preparation of recombinant viruses were essentially as described by Earl et al. [26]. Mammalian VLPs containing influenza HA were prepared by MVA vector infection of Vero cells and purified on 10–45% sucrose gradients as previously described [24]. DF-1 and Vero cells were originally obtained from ATCC (CRL-12203 and CCL-81, respectively). MDCK cells were obtained from Dr. Jacqueline Katz (CDC, Atlanta, GA, USA). All cells were maintained in Dulbecco’s modified Eagle medium supplemented with 10% FBS (HyClone, Logan, UT, USA), 2 mM l-glutamine, and 50 μg/ml gentamicin.

### Production of influenza B mAbs

Monoclonal Antibodies to influenza B HA were prepared by Precision Antibody (Columbia, MD) [27]. BALB/c mice were immunized and boosted with mammalian-derived influenza virus-like particles (VLPs) containing influenza B HA from either B/Brisbane/60/2008, B/Wisconsin/1/2010, or B/Massachusetts/02/2012. All mAbs were purified and concentrated by protein G chromatography for use in the experiments described.

### HA binding of mAb by ELISA

To screen mAbs for binding to influenza B HA, inactivated virus antigen (10 μg/ml) was used to coat 96 well Immulon-2HB plates (Dynex Technologies, Chantilly, VA, USA) overnight at 4°C. After blocking with 10% FBS in PBS, mAbs were serially diluted and incubated for 1 hour at 37°C. Goat anti mouse-HRP (KPL, Gaithersburg, MD, USA) at 1:5000 dilution was used for secondary detection and a 1:1 mixture of ABTS:H_2_O_2_ (Southern Biotech, Birmingham, AL, USA) was used as the substrate. Plates were read on a VersaMax microplate reader and data were generated with Softmax Pro (Molecular Devices, Sunnyvale, CA, USA). The endpoint titer was defined as the highest dilution that gave an absorbance value (405 nm) greater than 0.050.

### Hemagglutination inhibition (HI), plaque reduction neutralization, and HA-pseudotype neutralization assay

Assays to characterize the ability of mAbs to inhibit influenza virus hemagglutination or neutralization were performed as described previously [28]. The HI assay was performed in 96-well plates (U-bottom) by standard methods, using 0.5% chicken red blood cells suspended in PBS (pH 7.2). The plaque reduction neutralization assay was performed in MDCK cells with a 50:50 mixture of 2X EMEM plaque media (BioWhittaker, Walkersville, MD) and 2.4% Avicel RC-581 (FMC BioPolymer, Philadelphia, PA) in the presence of TPCK-trypsin (Sigma-Aldrich Corp. St. Louis, MO) at a final concentration of 2 μg/ml. Retroviral pseudotypes expressing the HA of influenza B were constructed and produced in 293T cells essentially as described previously [29, 30]. The antibody dilution causing a 95% reduction of vector expressed luciferase compared to control was used as the neutralization endpoint titer (IC_95_-neutralizing antibody titer).

### Selection of escape mutants

Isolation and selection of influenza virus escape mutants to monoclonal antibodies was performed in MDCK cells similar to previously reported procedures [28]. Influenza B virus was incubated with mAbs over a range of concentrations from 40–0.156 μg/ml in a total volume of 0.6 ml for 1 hour at room temperature, followed by infection of ~ 2.3 x 10^6^ MDCK cells with the virus-antibody mixtures. After 1.5 hour adsorption, virus inoculum was removed and the cells overlaid with serum-free media containing 2 μg/ml TPCK-trypsin. Using virus obtained at the highest concentration of mAb, the process was repeated at a 4-fold higher mAb concentration for up to 3 additional rounds of selection. Potential escape viruses were tested for reduced inhibition of neutralization by the mAb compared to the parent virus and consensus nucleotide sequences of viral HAs were determined by direct DNA-sequencing of RT-PCR products and compared with those of the parental virus.

### mAb capture potency ELISA

Purified mAbs were used to capture and quantify HA in vaccine samples [22]. Antibodies in PBS were used to coat 96-well immulon-2HB microplates overnight at 4°C, followed by washing and blocking with PBS/10% FBS. Reference antigen and vaccine samples were treated with 1% Zwittergent 3–14 for 30 minutes at room temperature as in the SRID assay, and then diluted with PBS to the desired starting concentration (minimum 10-fold dilution). Diluted samples were added to the microplate and serially diluted in PBS/10% FBS/0.05% Tween 20 (2-fold dilutions) and incubated for 2 hours at 37°C. The primary detection antibody was a purified rabbit polyclonal IgG, generated by the immunization of rabbits with plasmid DNA vectors expressing the lineage-specific influenza B HA and boosted with mammalian-derived VLPs containing the same influenza B HA. The secondary detection antibody was a goat anti-rabbit IgG conjugated with HRP. A 1:1 mix of ABTS:H_2_O_2_ was used as enzyme substrate. Plates were read on a VersaMax microplate reader and data were generated and analyzed with Softmax Pro. The HA concentration was determined by parallel line analysis of the four-parameter regression fits of test vaccine samples to that of the reference antigen standard included on each plate. Replicate test samples were included on each plate and replicate plates included in each assay. Assays were repeated a minimum of three times.

### Measurement of potency by SRID

SRID assays were performed essentially as originally reported [7, 8] with minor modifications made more recently [25, 31]. Vaccine potency was computed by the parallel line bioassay method using reference and test vaccine dose-response curves (log antigen dilution versus log zone diameter).

## Results

### Isolation and characterization of lineage-specific monoclonal antibodies to influenza B hemagglutinin

We generated mouse monoclonal antibodies (mAbs) to the hemagglutinin (HA) of recent influenza B vaccine strains from both B/Victoria and B/Yamagata lineages. Hybridoma clones secreting mAbs to influenza B HA were prepared by immunizing mice with mammalian-derived virus like particles (VLPs) [27] containing the HA of the influenza B Victoria lineage B/Brisbane/60/2008 virus (present in the vaccine since 2009) or the HA from either of two influenza B strains from the B/Yamagata lineage–B/Wisconsin/1/2010 (present in the vaccine 2012–2013) or B/Massachusetts/02/2012 (present in the vaccine 2013–2014). A sequential immunization scheme that utilized immunization with B/Brisbane/60/2008 (B/Victoria lineage) VLPs followed by immunization with B/Massachusetts/02/2012 (B/Yamagata lineage) VLPs and selection with HA antigens from both lineages was used to obtain antibodies that were cross-reactive with both influenza B lineages. Monoclonal antibodies were assessed for binding to their respective HA, as well as binding to HA from the alternate influenza B lineage, in an ELISA using inactivated virus. Antibodies were also evaluated for their ability to inhibit virus hemagglutination of chicken red blood cells and for virus neutralization activity. Table 1 lists mAbs from each group that showed strong binding to HA.

**Table 1:** Influenza B HA mAbs.

mAb	ELISA Titer (μg/ml)^1^	Hemagglutination Inhibition Titer (μg/ml)^2^		Pseudotype Neutralization Titer (μg/ml)^3^	
	Homologous Antigen	Victoria B/Brisbane/60/2008	Yamagata B/Texas/6/2011 or Homologous B/Yam	Victoria B/Brisbane/60/2008	Yamagata B/Wisconsin/1/2010
***B/Vic mAbs***					
**BR5A1** **4h8** **8F4**	3.04	1.10 2.47	>400	0.080	>20
**BR8E12**	3.04	2.47	>400	0.027	>20
**BR7B7**	3.04	1.56	>400	0.144	>20
***B/Yam mAbs***					
**WI3E6**	0.19	>400	0.874	>20	0.008
**WI3E8**	0.76	>400	0.874	>20	0.006
**MA1H4**	0.38	>400	0.39	>20	0.004
**MA3B2**	0.76	>400	1.56	>20	5.40
**MA3B8**	0.38	>400	0.78	>20	0.016
***Cross-reactive mAbs***					
**CR2F11**^**3**^	1.52	0.55	0.10	0.201	0.070
**CR2D5**	1.52	>400	>400	>20	>20

^1^ The end-point ELISA titer was defined as the lowest antibody concentration that gave an absorbance value at 405 nm greater than 0.05. ELISA binding was determined using B/Brisbane/60/2008 antigen for BR5A1, BR8E12, BR7B, CR2F11, and CR2D5; B/Wisconsin/1/2010 antigen for WI3E6 and WI3E8; and B/Massachusetts/02/2010 antigen for MA1H4, MA3B2, and MA3B8.

^2^ Hemagglutination inhibition (HI) titer was defined as the lowest antibody concentration that inhibited influenza B antigen agglutination of chicken red blood cells. B/Yamagata antigens used for HI were B/Texas/6/2011 for the B/Brisbane mAbs, B/Wisconsin/1/2010 for the B/Wisconsin mAbs, and B/Massachusetts/02/2010 for the B/Massachusetts and cross reactive mAbs.

^3^ Pseudotype neutralization titer was defined as the antibody concentration resulting in a 95% reduction in relative luciferase units of a retrovirus pseudotype expressing the indicated influenza B HA.

The mAbs selected against the B/Victoria virus B/Brisbane/60/2008 showed similar levels of binding to inactivated B/Brisbane antigen when measured in ELISA and had similar hemagglutination inhibition (HI) titers against B/Brisbane virus (Table 1) as well as another tested B/Victoria virus, B/Malaysia/2506/2004 (data not shown). None had HI activity against the B/Yamagata virus B/Texas/6/2011 (a B/Wisconsin/1/2010-like virus). Further characterization of these mAbs using a pseudotype neutralization assay indicated effective neutralization of B/Brisbane/60/2008 HA-containing pseudotype virus with no significant difference among their titers. As in the ELISA and HI assays, none of the B/Brisbane mAbs had appreciable activity against a B/Yamagata HA-containing pseudotype virus, confirming their lineage specificity.

Similar characterizations were performed for the B/Yamagata mAbs selected against B/Wisconsin/1/2010 and B/Massachusetts/2/2012 (Table 1). All of the B/Yamagata mAbs had strong binding and HI activity to their homologous inactivated B/Yamagata antigen (either B/Wisconsin or B/Massachusetts), as well as HI activity to other B/Yamagata strains (data not shown). When tested in the pseudotype neutralization assay, these mAbs neutralized pseudotype virus expressing the HA of the B/Yamagata strain B/Wisconsin/1/2010, but not pseudotype virus expressing the HA from B/Brisbane/60/2008.

In contrast to these influenza B lineage-specific mAbs, cross-reactive mAbs CR2F11 and CR2D5 bound antigens from each lineage in ELISAs (Table 1 and data not shown). One of these cross-reactive mAbs, CR2D5, did not have either HI or pseudotype neutralization activity against any tested HAs. However, the other cross-reactive mAb, CR2F11, had HI activity and pseudotype neutralization activity against both lineages of influenza B (Table 1).

### Epitope analysis of influenza B lineage-specific mAbs

HA epitopes recognized by the influenza B mAbs were determined by generating escape mutant viruses to the selected antibodies. The escape mutants were isolated by culturing virus in the presence of serial dilutions of mAbs followed by infection of MDCK cells with the virus-antibody mixtures. Virus was harvested from the highest concentration of mAb and the process repeated at a four-fold higher concentration of the same mAb. After several rounds of selection, the HA gene of each potential escape virus was sequenced and aligned to the parent virus used for selection.

**Fig 1:**
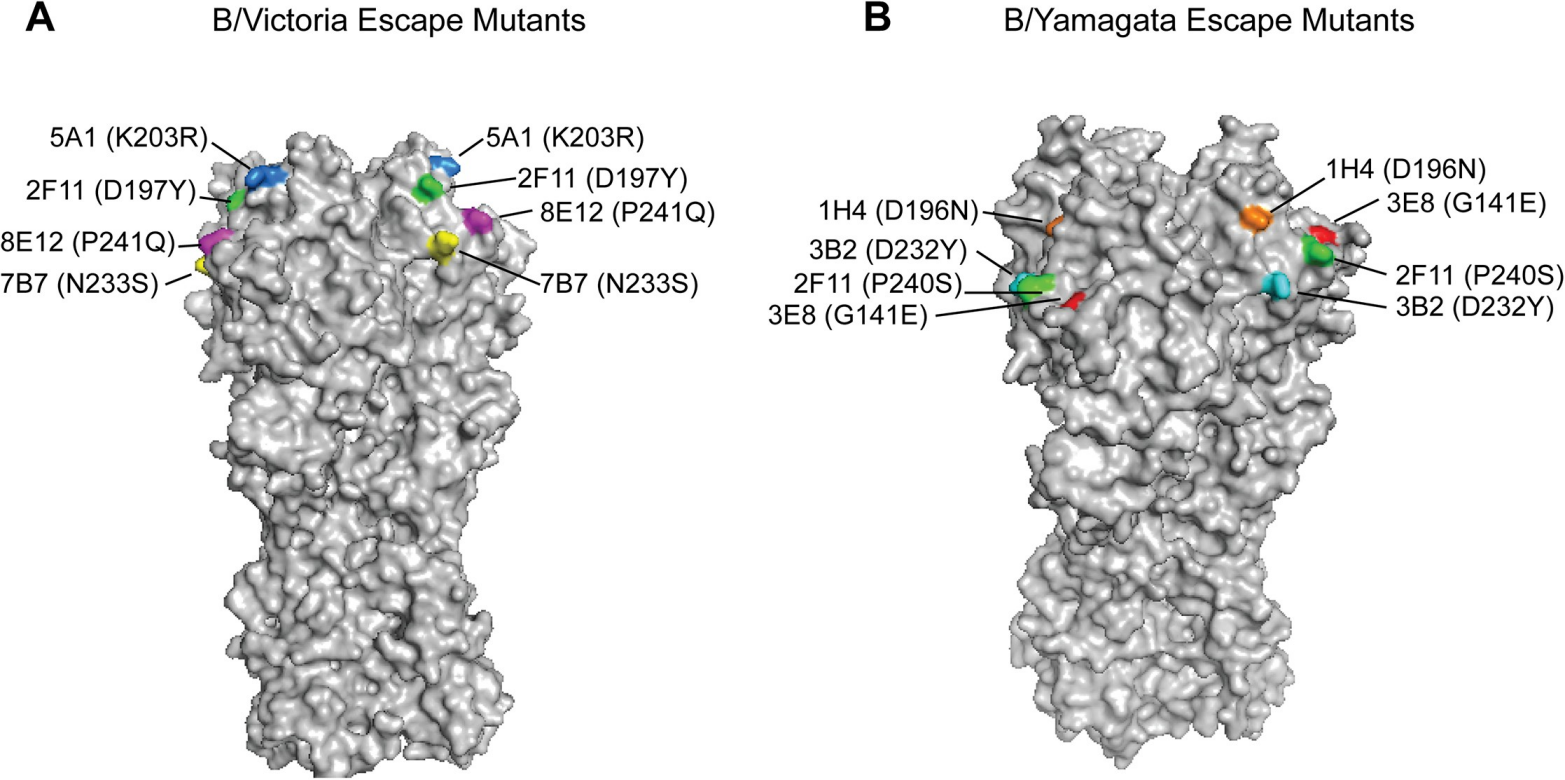
Location of HA amino acid changes in influenza B escape mutants. (A) Antigenic structure of the B/Victoria lineage HA trimer (B/Brisbane/60/2008 PDB ID: 4FQM) and location of the escape mutants to mAbs BR5A1 (blue), BR8E12 (magenta), BR7B7 (yellow), and CR2F11 (green). (B) Antigenic structure of the B/Yamagata lineage HA trimer (B/Yamanashi/166/1998 PDB ID: 4M40) and location of the escape mutants to mAbs MA1H4 (orange), WI3E8 (red), MA3B2 (cyan), and CR2F11 (green). Escape mutants to the cross-reactive mAb CR2F11 were derived from both B/Victoria and B/Yamagata viruses.

Escape mutant viruses with single amino acid changes were obtained for the three B/Brisbane/60/2008 mAbs BR5A1, BR8E12, and BR7B7. The BR5A1 escape mutant (5A1v) had an amino acid substitution at position 203 from lysine to arginine (amino acid numbering throughout the text refers to the mature HA, excluding the HA N-terminus signal peptide), 8E12v had a proline to glutamine substitution at position 241, and 7B7v had an asparagine to serine substitution at position 233 (Fig 1A). These amino acid changes are near the 190 helix and the receptor-binding domain of HA, and this region of the HA has previously been proposed to be a major influenza B antigenic site that is specific for B/Victoria viruses [32]. In addition, a B/Brisbane escape mutant was generated to cross-reacting mAb CR2F11 and this virus had an amino acid substitution at position 197 from aspartic acid to tyrosine.

To further characterize the epitopes identified by the B/Brisbane mAbs and their potential overlap, each mAb was evaluated for its ability to neutralize B/Brisbane and the B/Brisbane escape mutant viruses in a plaque-reduction neutralization assay (Table 2). As expected, each mAb neutralized the parent B/Brisbane virus, but not the escape virus generated to itself, and each mAb neutralized the escape virus generated to the cross-reactive mAb CR2F11. The mAb BR5A1 neutralized the other escape viruses, suggesting that the epitope identified by BR5A1 was distinct from those identified by the other mAbs. On the other hand, mAb BR8E12 did not neutralize the BR5A1 escape virus. Taken together, these results suggest that the epitopes recognized by BR5A1 and BR8E12 may be overlapping but at least partially distinct. The N233S mutation in the BR7B7v escape virus results in the loss of a potential glycosylation epitope on the HA, and thus may not reflect the actual binding site of the BR7B7 mAb. Since BR7B7 does not neutralize either 5A1v or 8E12v escape viruses, its epitope probably overlaps those of BR5A1 and BR8E12. Nevertheless, since BR7B7 is the only mAb unable to neutralize the 7B7v escape virus, it is different from the other B/Brisbane mAbs. Finally, cross-reactive mAb CR2F11, which neutralizes both B/Victoria and B/Yamagata viruses, did not neutralize either 5A1v or 8E12v escape viruses.

**Table 2:** mAb neutralization of B/Victoria escape mutants.

Virus^1^	mAb^2^			
	BR5A1	BR8E12	BR7B7	CR2F11
**B/Brisbane**	**+**	**+**	**+**	**+**
**5A1v (K203R)**	**-**	**-**	**-**	**-**
**8E12v (P241Q)**	**+**	**-**	**-**	**-**
**7B7v (N233S)**	**+**	**+**	**-**	**+**
**2F11v (D197Y)**	**+**	**+**	**+**	**-**

^1^ Each virus was titrated and diluted to approximately 500 pfu/ml and incubated with mAb concentrations from 80 to 0.31 μg/ml for incubation with mAb.

^2^ Each mAb completely neutralized B/Brisbane at concentrations between 1.25–5 μg/ml (scored + in the Table); escape mutants requiring a 10-fold or greater concentration of mAb for neutralization (relative to B/Brisbane neutralization) were scored negative (resistant to mAb neutralization).

Analysis of escape mutant viruses generated to B/Yamagata mAbs revealed that two distinct epitopes were recognized by the mAbs (Fig 1B). The WI3E8 escape mutant (3E8v) had an amino acid substitution at position 141 from glycine to glutamic acid. This amino acid change is near the 150 loop and this region of the HA has previously been proposed to be a major influenza B antigenic site [33] that is specific for B/Yamagata viruses [34]. Other escape mutations were identified near the 190 helix and included a D196N for the MA1H4 escape mutant (1H4v) and a D232Y for the MA3B2 escape mutant (3B2v). Interestingly, the D196N substitution adds back a potential glycosylation motif that is typically lost from influenza B viruses during egg adaptation [35]. In addition, a B/Mass escape mutant was generated to cross-reacting mAb CR2F11 and this virus had a proline to serine substitution at position 240.

Further characterization of the epitopes identified by the B/Yamagata mAbs and their potential overlap was evaluated in plaque-reduction neutralization assays (Table 3). Each mAb neutralized the B/Massachusetts virus, but not the escape virus generated to itself. The mAb WI3E8 and WI3E6 were unable to neutralize the escape virus generated to mAb WI3E8, but were able to neutralize all of the other B/Yamagata escape viruses, indicating that the epitope recognized by WI3E8 and WI3E6 was overlapping and distinct from epitope(s) identified by the other B/Yamagata mAbs. Furthermore, the other B/Yamagata mAbs (MA1H4, MA3B2, and MA3B8) were able to neutralize the 3E8v escape virus. The escape mutant generated to MA1H4 had a D196N substitution, which adds back a potential glycosylation motif, and thus may not reveal the actual binding site for mAb MA1H4. Nevertheless, the mAbs MA3B8 and MA3B2 were also unable to neutralize this escape virus, suggesting that this mutation affected binding of MA1H4, MA3B8, and MA3B2 similarly, whereas WI3E8 and WI3E6 both neutralized the 1H4v escape virus. Finally, the escape mutant virus to MA3B2 was poorly neutralized by MA3B2, MA3B8, and MA1H4, again suggesting that all three recognize the same or overlapping epitopes. The mAbs WI3E8 and WI3E6, however, neutralized the 3B2v escape virus, indicating a distinct epitope. The cross-reacting mAb CR2F11 neutralized the 3E8v escape virus, but not the 1H4v or 3B2v escape viruses.

**Table 3:** mAb neutralization of B/Yamagata escape mutants.

Virus^1^	mAb					
	WI3E8	WI3E6	MA1H4	MA3B2	MA3B8	CR2F11
**B/Massachusetts**	**+**	**+**	**+**	**+**	**+**	**+**
**3E8v (G141E)**	**-**	**-**	**+**	**+**	**+**	**+**
**1H4v (D196N)**	**+**	**+**	**-**	**-**	**-**	**-**
**3B2v D232Y)**	**+**	**+**	**-**	**-**	**-**	**-**
**2F11v P240S)**	**+**	**+**	**+**	**+**	**+**	**-**

^1^ Plaque reduction assays and criteria for scoring neutralization or resistance to neutralization are described in the legend to Table 1.

### Monoclonal antibody identity assays that distinguish the influenza B lineage components in inactivated influenza vaccines

**Fig 2:**
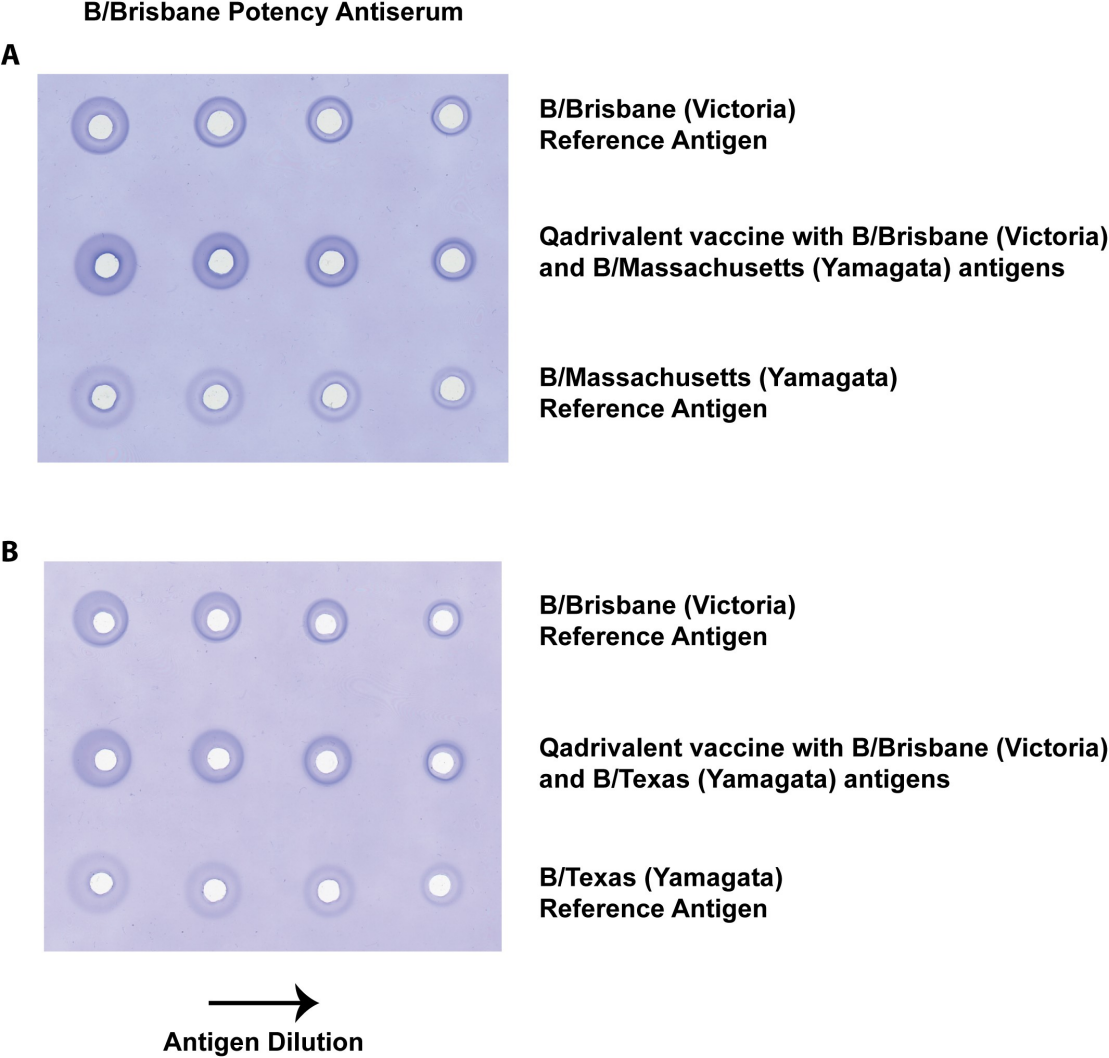
SRID analysis of quadrivalent influenza vaccines using sheep polyclonal antiserum produced to B/Brisbane/60/2008. Dilutions of quadrivalent influenza vaccine containing B/Brisbane/60/2008 and B/Massachusetts/2/2012 (A) or B/Brisbane/60/2008 and B/Texas/02/2013 (B) were loaded onto agarose gels (rows 2) along with dilutions of the two corresponding reference antigens (rows 1 and 3) and analyzed by standard SRID using B/Brisbane/60/2008 reference antiserum (Lot B-Ab-1108).

The single-radial immunodiffusion (SRID) assay that is used for potency determination of inactivated influenza vaccines is also typically used as an identity test to verify the HA antigens included in formulated trivalent and quadrivalent vaccines. Since the two lineages of influenza B are antigenically more closely related than subtypes of influenza A, there is a potential problem in distinguishing the influenza B components in quadrivalent vaccines containing two influenza B antigens. Fig 2 shows SRID gels that used the sheep polyclonal potency antiserum to B/Brisbane/60/2008 (Victoria lineage) to assay quadrivalent vaccines containing B/Brisbane/60/2008 and either B/Massachusetts/02/2012 (Fig 2A) or B/Texas/6/2011 (Fig 2B). The B/Brisbane potency antiserum shows cross-reactivity to both the B/Massachusetts and B/Texas Yamagata lineage Reference Antigens. While good precipitin rings are produced for each quadrivalent vaccine analysis with the B/Brisbane potency antiserum (2^nd^ row, Fig 2A and 2B), the cross-reactivity of the antiserum with the two Reference Antigens from the influenza B Yamagata lineage makes verification of identity in the vaccine problematic.

**Fig 3:**
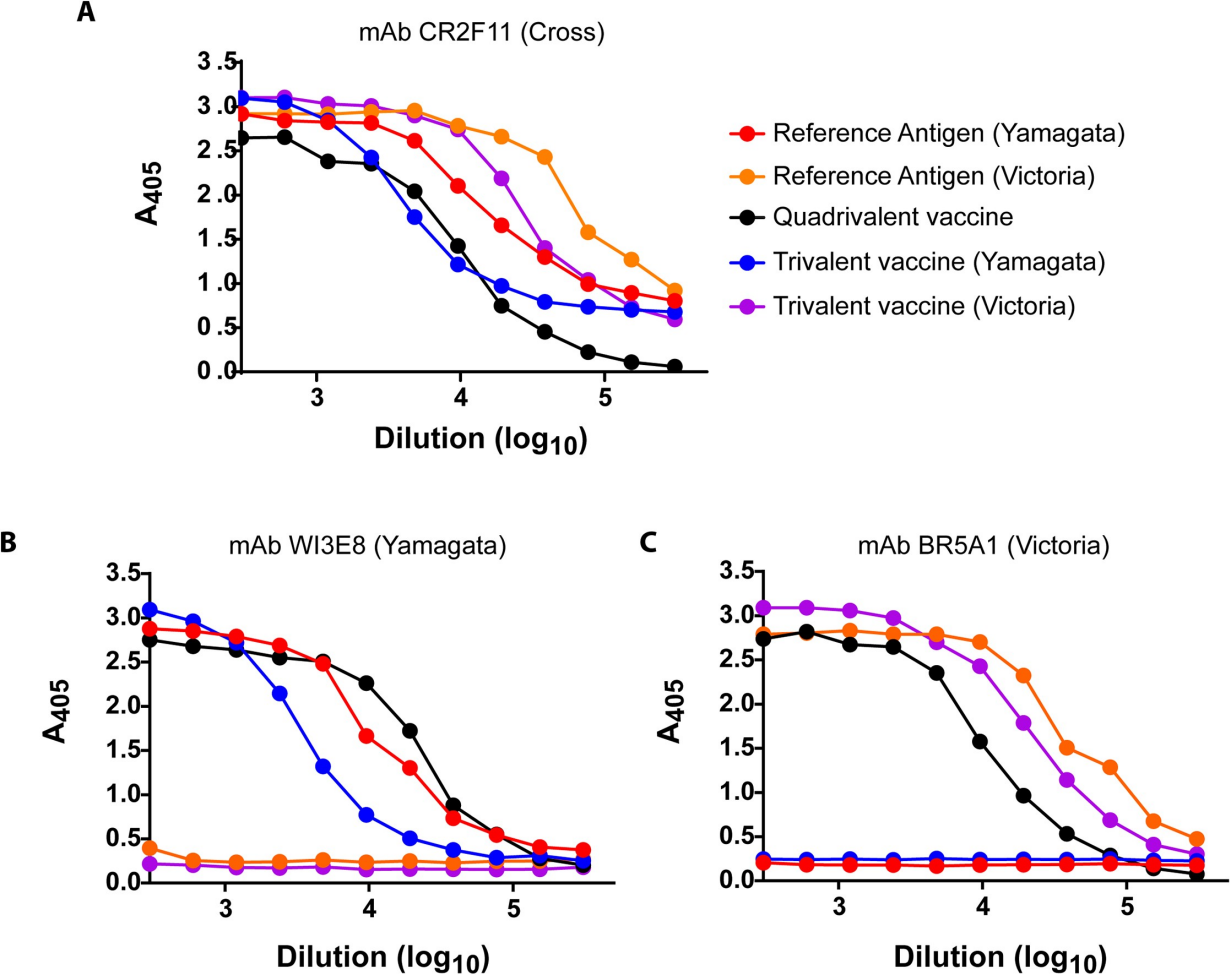
ELISA identity analysis of trivalent and quadrivalent influenza vaccines using lineage-specific mAbs. ELISA plates were coated with the indicated purified mAbs at 2 μg/ml and used to capture Reference Antigens B/Brisbane/60/2008 (60 μg/ml) and B/Massachusetts/2/2012 (58 μg/ml), a quadrivalent vaccine containing B/Brisbane and B/Mass antigens at 24 and 40 μg/ml, respectively, a trivalent vaccine containing B/Florida/4/2006 (25 μg/ml), and a trivalent vaccine containing B/Brisbane (27 μg/ml). The starting dilution for all reference antigens and vaccines was 1:300.

An alternative identity test for the influenza B component of inactivated vaccines was developed using lineage-specific mAbs as capture antibodies in an ELISA format (Fig 3). As shown in Fig 3A, the cross-reactive mAb CR2F11 easily captured HA from B/Victoria and B/Yamagata Reference Antigen, trivalent vaccines containing either a B/Victoria or B/Yamagata component, and a quadrivalent vaccine containing antigen from both lineages. On the other hand, a B/Yamagata-specific mAb (WI3E8) captured B/Massachusetts Reference Antigen and trivalent and quadrivalent vaccines containing B/Yamagata antigens, but B/Victoria Reference Antigens and trivalent vaccines with B/Victoria antigens were not bound (Fig 3B). Similarly, the B/Brisbane/60/2008 Reference Antigen and B/Brisbane-containing trivalent and quadrivalent vaccines were bound well when a B/Victoria-specific mAb (BR5A1) was used as the capture mAb, but there was no binding of a trivalent vaccine containing a B/Yamagata antigen or its associated Reference Antigen (Fig 3C). These results demonstrate the feasibility of using lineage-specific mAbs as identity tests to verify the influenza B component in inactivated vaccines.

**Fig 4:**
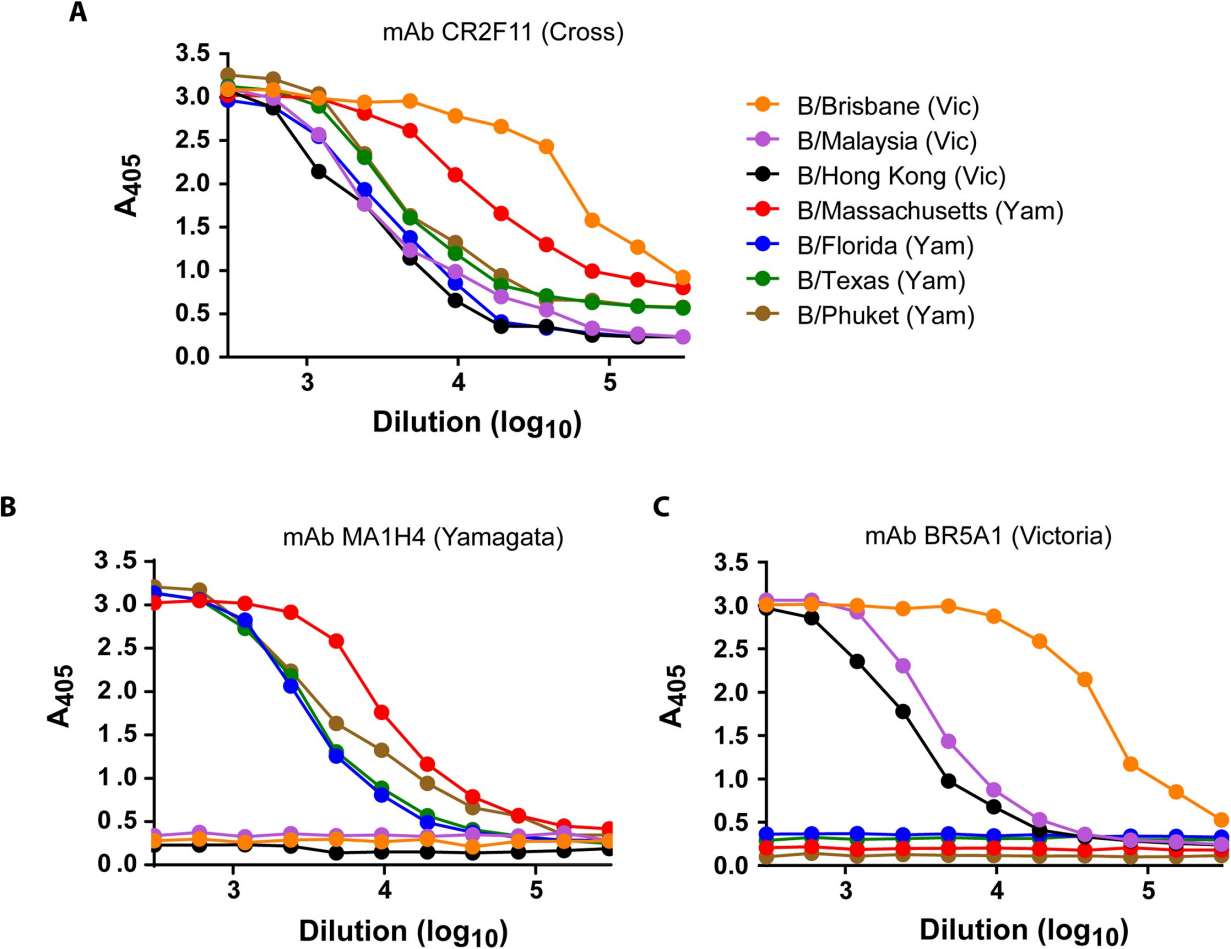
Lineage specificity of mAb-capture ELISA. ELISA plates were coated with the indicated purified mAbs at 2 μg/ml and used to capture Reference Antigens B/Brisbane/60/2008 (60 μg/ml), B/Malaysia/2506/2004 (76 μg/ml), B/Hong Kong/330/2001 (69 μg/ml), B/Massachusetts/2/2012 (58 μg/ml), B/Florida/4/2006 (79 μg/ml), B/Texas/6/2011 (80 μg/ml), and B/Phuket/3073/2013 (78 μg/ml). The starting dilution for all reference antigens was 1:300.

Since influenza B antigens in the vaccines change periodically, we tested the lineage-specific mAbs for their ability to bind Reference Antigens for recent influenza B vaccine strains. The cross-reactive mAb CR2F11 bound all of the tested Reference Antigens well (Fig 4A). In contrast, B/Victoria Reference Antigens Brisbane/60/2008 (vaccine component since 2009), B/Malaysia/2506/04 (vaccine component 2006–2008) and B/Hong Kong/330/01 (vaccine component 2002–2004) showed binding only to the B/Victoria BR5A1 mAb in an ELISA-based identity assay and not to the Yamagata lineage MA1H4 mAb (Fig 4C). Similarly, B/Phuket/3073/2013 (2015–2016), B/Massachusetts/02/2012 (2013–2015), B/Texas/6/2011 (2012–2013), and B/Florida/4/2006 (2008–2009) Reference Antigens bound to the B/Yamagata MA1H4 mAb, but not to the BR5A1 mAb (Fig 4B). Similar results were obtained using other mAbs from the Victoria and Yamagata antibody panels (data not shown). These results confirmed the lineage-specificity of the mAbs and suggested that identity tests using lineage-specific mAbs would not necessarily have to be updated with every influenza B vaccine strain change.

### ELISA-based potency assays using lineage-specific influenza B mAbs

**Fig 5:**
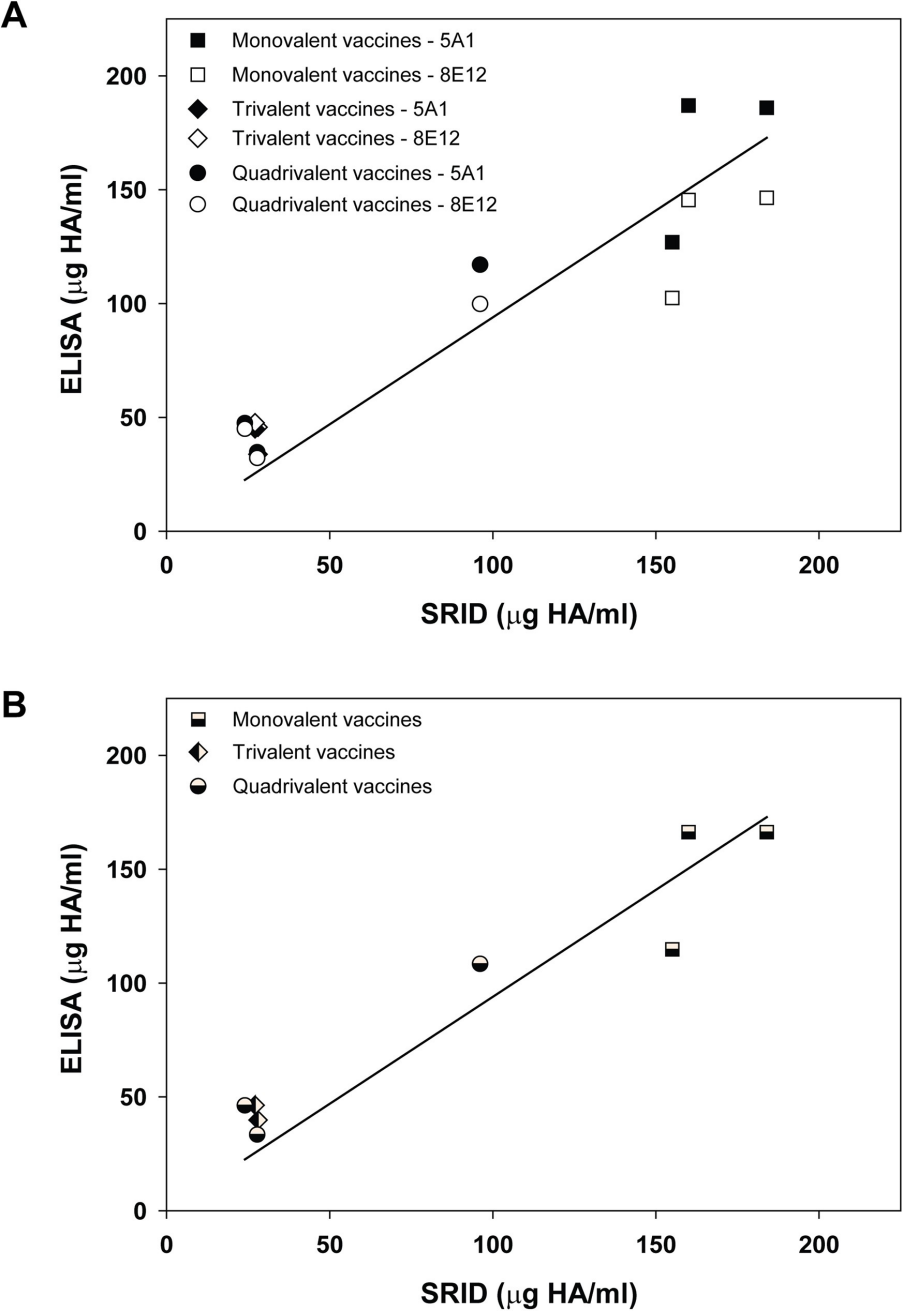
Correlation between SRID potency values and ELISA potency values determined for vaccines containing B/Victoria antigens. (A) Eight vaccine samples, including 3 monovalent vaccines (■), 2 trivalent vaccines (▲), and 3 quadrivalent vaccines (●) were analyzed for HA content by standard SRID and mAb-capture ELISA using B/Victoria mAbs BR5A1 (closed symbols) and BR8E12 (open symbols). (B) SRID potency values for each vaccine plotted against the mAb-capture ELISA potency value using the combined (mean) BR5A1 and BR8E12 ELISA potency values.

Lineage-specific mAbs were used as capture antibodies for influenza HA in a previously described ELISA format [22] to quantify HA in vaccine samples. Since the ideal number of mAbs needed for development of a reliable mAb-based ELISA potency assay is still unknown, we used two mAbs, representing different HA epitopes, for all ELISA potency measurements. B/Victoria mAbs BR5A1 and BR8E12 were used to measure the HA content in monovalent, trivalent, and quadrivalent vaccines containing the B/Victoria antigen B/Brisbane/60/2008 by comparing the binding of HA in vaccine samples relative to a reference antigen standard with an assigned HA content. A total of eight different vaccine samples, supplied from three different manufacturers, were analyzed. Traditional SRID potency assays were run in parallel for each vaccine sample using the same reference standard for monovalent and trivalent vaccine samples. SRID analysis of the quadrivalent vaccine samples used a bivalent reference standard containing equal amounts of each influenza B Reference Antigen to correct for cross-reactivity of the potency antiserum in the SRID assay (e.g., Fig 2). This modification to the SRID assay is necessary for accurate measurement of HA in quadrivalent influenza vaccines. The ELISA and SRID potency values were compared and the correlation plotted (Fig 5). In general, the ELISA potency values determined using each B/Victoria mAb were similar and there was no obvious pattern to the differences measured for the various vaccine samples, other than the somewhat wider monovalent vaccine ELISA values determined by the two mAbs (Fig 5A). Higher variability for monovalent vaccine samples may be partially related to the high HA concentration of these samples, which requires large dilutions of the samples before assay. Overall however, the BR5A1 ELISA potency values were higher than those determined using BR8E12 in 6 of the 8 analyses, by an average of approximately 18%. In the 2 analyses in which the BR8E12 ELISA potency values were higher, the average difference was approximately 20%. Fig 5B shows the correlation between the SRID values for each vaccine sample compared to the mAb ELISA when the values determined using the two mAb are combined and averaged. The SRID and mAb ELISA potency values correlated well over a wide range of HA antigen values in the vaccine samples with a regression fit slope of 0.94.

**Fig 6:**
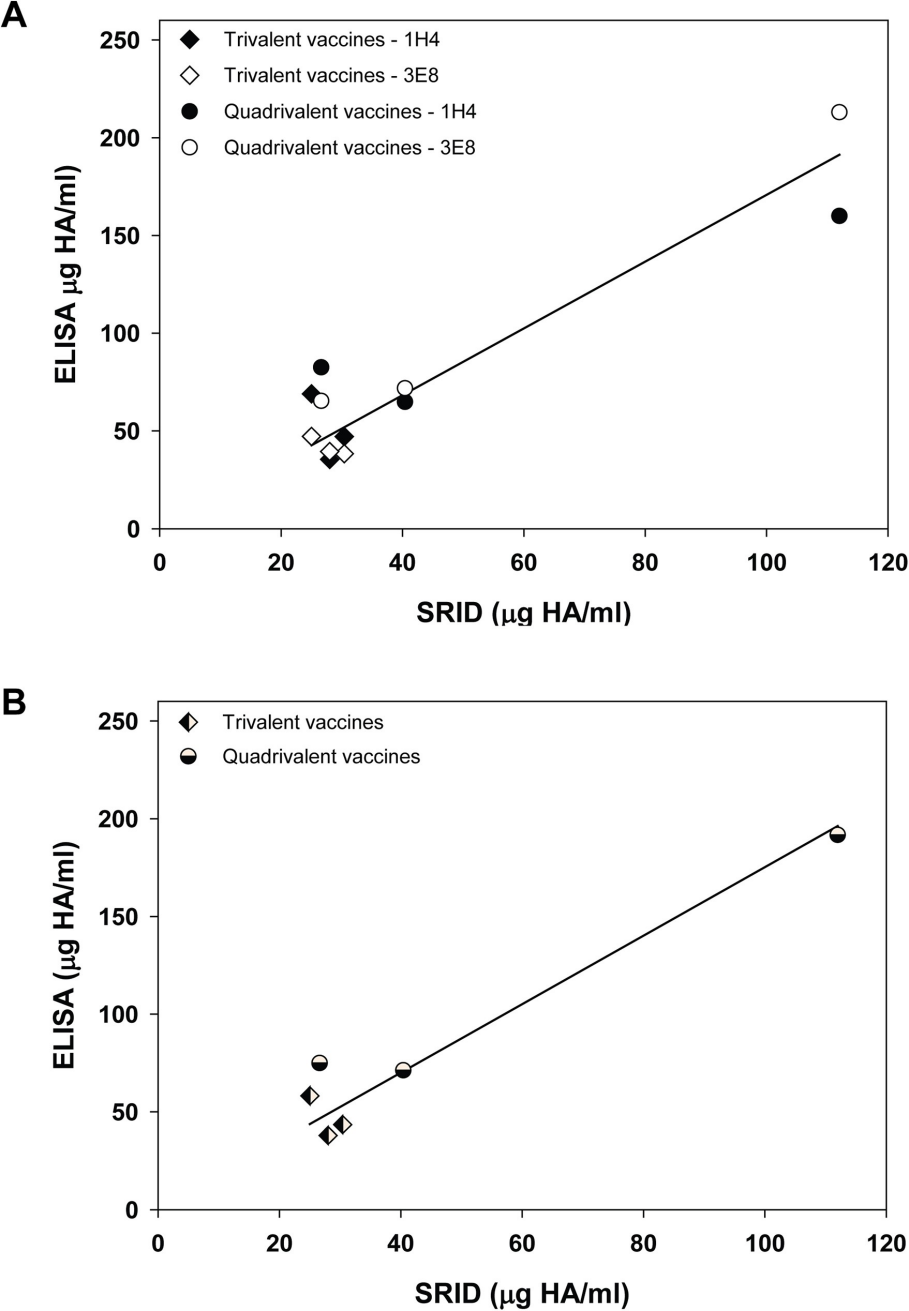
Correlation between SRID potency values and ELISA potency values determined for vaccines containing B/Yamagata antigens. (A) Six vaccine samples, including 3 trivalent vaccines (▲) and 3 quadrivalent vaccines (●) were analyzed for HA content by standard SRID and mAb-capture ELISA using B/Yamagata mAbs MA1H4 (closed symbols) and WI3E8 (open symbols). (B) SRID potency values for each vaccine plotted against the mAb-capture ELISA potency value using the combined (mean) MA1H4 and WI3E8 ELISA potency values.

Fewer vaccine samples containing B/Yamagata antigens were available for analysis, but interestingly these vaccines contained three different B/Yamagata antigens. Whereas all of the vaccines containing a B/Victoria antigen contained the same B/Brisbane antigen, three trivalent vaccines contained B/Florida/4/2006 as the influenza B/Yamagata antigen, two of the quadrivalent vaccines contained B/Massachusetts/02/2012 as the influenza B/Yamagata component, and B/Texas/6/2011 was the B/Yamagata antigen in the third quadrivalent vaccine. B/Yamagata mAbs MA1H4 and WI3E8 were used to measure the influenza B HA antigen content in all vaccines using the capture ELISA format and the results were compared to potency values obtained by SRID analyses conducted in parallel on the same samples (Fig 6). There was general agreement between the ELISA potency values obtained using the two B/Yamagata mAbs. For 3 vaccine samples, values were higher with mAb MA1H4 by an average of 33%; for the other 3 vaccine samples, mAb WI3E8 values were higher by an average of 18% (Fig 6A). As for the B/Victoria antigen analyses, there was a general correlation between the SRID and mAb ELISA values determined for the six B/Yamagata-containing vaccine samples, again over a fairly wide range of HA antigen content. But, the ELISA potency values were uniformly higher than those determined by SRID for all B/Yamagata antigens as indicated by the regression fit slope of 1.75 (Fig 6B).

### Comparison of ELISA and SRID potency of temperature-stressed vaccines

We used heat treatment to accelerate the decline in vaccine potency in order to evaluate whether the mAb capture ELISA was able to distinguish stressed, i.e., sub-potent, from unstressed vaccine samples. Aliquots of a quadrivalent vaccine sample containing influenza B antigens B/Brisbane (B/Victoria) and B/Texas (B/Yamagata) were incubated at 55°C for 0.5, 1, 4, and 24 hours, and then the potency of each B antigen was determined by mAb capture ELISA and SRID relative to the vaccine sample maintained at 4°C (Table 4). As in the previous ELISA analyses, two mAbs from each lineage were used to capture and quantify antigen. Heat treatment rapidly reduced the vaccine potency of each influenza B antigen in the vaccine sample as measured by SRID. Similar declines in potency of each antigen were measured in the capture ELISA with all of the mAbs, demonstrating that the influenza B mAb capture ELISA is capable of distinguishing heat-stressed from unstressed vaccines and is similar to the SRID for quantifying the resultant loss of potency.

**Table 4:** SRID and ELISA influenza B potency of influenza quadrivalent vaccine subjected to temperature stress at 56°C.

	B/Victoria Antigen Potency (% Unstressed Potency)			B/Yamagata Antigen Potency (% Unstressed Potency)		
Time at 56°C	B/Bris SRID	mAb BR5A1	mAb BR8E12	B/Texas SRID	mAb MA1H4	mAb WI3E8
**0**	100	100	100	100	100	100
**0.5**	43	56	51	43	39	40
**1**	27	42	42	27	20	27
**4**	5.3	4.2	4.3	<1	<1	<1
**24**	2.7	<1	<1	<1	<1	<1

## Discussion

Co-circulation of two distinct lineages of influenza B has led to the development of quadrivalent influenza vaccines that contain two influenza B antigens. Indeed, prior to the development of quadrivalent vaccines, influenza B lineage mismatches between the vaccine components and circulating viruses occurred relatively frequently [36]. There are now several quadrivalent influenza vaccine available [5]. The results from numerous clinical studies have demonstrated that immunogenicity to antigens in a trivalent vaccine containing two influenza A components (H1N1 and H2N3) and one influenza B antigen is not compromised by the addition of a second influenza B antigen and there is superior immunogenicity to the additional influenza B antigen [37]. In addition, several analyses have now shown the cost-effectiveness of seasonal quadrivalent versus trivalent vaccination, including studies in the United States [38]. Nevertheless, development of quadrivalent inactivated influenza vaccines highlighted some of the challenges in seasonal influenza vaccine production such as the lack of optimal high growth influenza B vaccine candidate viruses and the production and distribution of another set of reagents for standardization of vaccine potency. Further, the potential for cross-reactivity of the influenza B reagents necessitated modifications in the standard SRID potency assay, which is used for both identity and potency determination of inactivated influenza vaccines. Currently, the polyclonal sheep antisera used in the assay is routinely screened for cross-reactivity for the two influenza B lineages, and the SRID assay is modified for use with quadrivalent vaccines by formulation of a bivalent reference standard containing equal amounts of each influenza B Reference Antigen.

Lineage-specific mAbs, such as those described here, may provide another option for use in the influenza B antigen identity testing that is required for vaccine evaluation. The absence of cross-reactivity of mAbs specific for each lineage provides unambiguous verification of antigen identity in vaccine samples, including quadrivalent vaccines. Encouragingly, the lineage-specific mAbs isolated and characterized in this study bound well to the HA of influenza B strains included in influenza vaccines over a period of more than 10 years. Also, some of the mAbs are similar to influenza B mAbs previously reported, including antibodies that recognize B/Victoria epitopes at position 241 and B/Yamagata epitopes at 141 [32, 34], and which pre-date the vaccines tested in our experiments. Taken together, this suggests that some epitopes specific to the two influenza B HAs have not changed in a very long time and that reagents to distinguish them might not need frequent updating.

Influenza HA mAbs have been used as capture antibodies in several potency assays being developed as possible alternatives to the SRID assay [19–22]. While the SRID assay has many strengths, including experience gained over several decades of use with multiple vaccines produced from various manufacturers, there are limitations to the assay that might be addressed with more modern techniques. These strengths and limitations of the SRID have been noted in a recent review [14]. The lineage-specific influenza B HA mAbs described in the current study are a source of reagents that can be used for further development of antibody-capture alternative potency assays. The results indicated that HA could be quantified in a variety of vaccine samples, including monovalent bulks, trivalent vaccines, and quadrivalent vaccines, from multiple manufacturers, over a wide range of HA content. The HA values determined by both sets of lineage-specific mAb ELISAs generally correlated with SRID potency values run in parallel, although the values were not always equivalent. In a previous study, the use of more than one mAb for capture in the potency ELISA appeared to provide a better correlation with the SRID potency assay results when the results of all of the potency assay results were combined [22]. Although only two mAbs were used in each ELISA potency assays in the current study, a better correlation with SRID was observed in most cases by combining the two mAb-ELISA results (Figs 5 and 6). Nevertheless, additional studies will be needed to determine how many mAbs are needed for development of a reliable alternative potency assay, as well as to define the characteristics of the antibodies for optimal assay set-up. In addition to the possibility of improving assay robustness, the availability of mAbs to different HA epitopes may provide assay redundancy that mitigates the effect of epitope drift on the assay set-up.

Importantly however, the results in the present study demonstrated that mAb-capture assays are capable of quantifying HA content of two different influenza B antigens in the same quadrivalent vaccine. The availability of multiple mAbs to the HA of each lineage, and the capability of the mAbs to recognize influenza B HAs over a wide range of years, further suggests the overall feasibility of an antibody capture approach to influenza B HA determination. In addition, in a very limited evaluation of the CR2F11 mAb as a capture mAb in the potency ELISA, results indicated that such antibodies might also be useful for analysis of monovalent vaccine bulks (data not shown). Finally, the results indicated that both sets of lineage-specific mAbs were stability-indicating when vaccine samples were subjected to heat stress. The relative potency decline measured by SRID and the mAb-capture ELISA were comparable.

Correlation, but not always equivalence, between SRID potency and potency determined by alternative methods has been observed previously with most antibody-based assays, including our earlier work with influenza A H1N1 vaccines [22]. Our results in the present study indicated a relatively better correlation between SRID and B/Victoria mAb ELISA values than between the SRID and B/Yamagata mAb ELISA values. The reason for this is not clear, but it is possible that it is related to the differences between the antigen standard (whole inactivated influenza virus) and the vaccine (detergent split, subunit antigen). Monoclonal antibodies in a capture format and polyclonal antibodies in an SRID gel format may not always interact with the different presentations of HA in the same manner. Other studies have addressed this issue by preparing a working standard that is similar in form to the vaccines being tested and calibrated by SRID to the whole inactivated influenza virus Reference Standard provided by regulatory agencies [19, 20]. It is not known whether this approach would provide a standard applicable to all types of vaccines or whether manufacturer-specific working standards would be needed. Alternatively, the possibility that new potency assays for influenza vaccines may need clinical trials to verify that potency determined by an alternative assay predicts vaccine efficacy has been proposed [39]. Clearly, additional studies will be needed to resolve such issues and determine the best way forward for development and implementation of alternative potency assays for influenza vaccines.
